# Exposure of the U.S. Population to Acrylamide in the National Health and Nutrition Examination Survey 2003–2004

**DOI:** 10.1289/ehp.0901021

**Published:** 2009-10-14

**Authors:** Hubert W. Vesper, Samuel P. Caudill, John D. Osterloh, Tunde Meyers, Deanna Scott, Gary L. Myers

**Affiliations:** Division of Laboratory Sciences, National Center for Environmental Health, Centers for Disease Control and Prevention, Atlanta, Georgia, USA

**Keywords:** acrylamide, biomonitoring, glycidamide, human exposure, hemoglobin adducts, NHANES, U.S. population

## Abstract

**Background:**

The lifelong exposure of the population to acrylamide has raised concerns about the possible health effects of the chemical. Data on the extent of exposure to acrylamide and its primary metabolite, glycidamide, are needed to aid in the assessment of potential health effects.

**Objectives:**

The aim of this study was to assess human exposure to acrylamide and glycidamide in the general U.S. population through the measurement of hemoglobin adducts of acrylamide (HbAA) and glycidamide (HbGA).

**Methods:**

HbAA and HbGA were measured in 7,166 subjects from the National Health and Nutrition Examination Survey. Stratified HbAA and HbGA data were reported by sex, age groups, race/ethnicity (Mexican American, non-Hispanic black, non-Hispanic white), and smoking status based on serum cotinine levels. Covariate-adjusted geometric means for each demographic group were calculated using multiple regression analysis.

**Results:**

HbAA and HbGA levels ranged from 3 to 910 and from 4 to 756 pmol/g hemoglobin, respectively, with smokers having the highest levels overall. Tobacco smoke exposure in nonsmokers had a small but significant effect on HbAA and HbGA levels. Adjusted geometric mean levels for children 3–11 years of age were higher than for adults ≥ 60 years of age [mean (95% confidence interval): HbAA, 54.5 (49.1–51.5) and HbGA, 73.9 (71.3–76.6) vs. HbAA, 46.2 (44.3–48.2) and HbGA, 41.8 (38.7–45.2)]. Levels were highest in Mexican Americans [HbAA: 54.8 (51.9–57.8), HbGA: 57.9 (53.7–62.5)], whereas non-Hispanic blacks had the lowest HbGA levels [43.5 (41.1–45.9)].

**Conclusions:**

U.S. population levels of acrylamide and glycidamide adducts are described. The high variability among individuals but modest differences between population subgroups suggest that sex, age, and race/ethnicity do not strongly affect acrylamide exposure. Adduct concentration data can be used to estimate relative exposure and to validate intake estimates.

Acrylamide is an environmental and industrial chemical. People are exposed to this chemical through its formation in foods during heating (Dybing et al. 2005; Mottram et al. 2002; Stadler et al. 2002), tobacco smoke (Smith et al. 2000), and occupational activities involving the production and use of acrylamide (Hagmar et al. 2005; Jones et al. 2006; Paulsson et al. 2006). The health effects of acrylamide have been reviewed extensively in the literature (Exon 2006; Klaunig 2008; LoPachin et al. 2008; Shipp et al. 2006). Acrylamide can be neurotoxic in humans and animals and carcinogenic in animals, and has been categorized as a suspected human carcinogen [Hagmar et al. 2001; International Agency for Research on Cancer (IARC) 1995; Manjanatha et al. 2006]. It is also a reproductive and developmental toxicant in male rats and mice [National Toxicology Program (NTP) 2004]. The lifelong exposure of most of the population to acrylamide through food and smoking has raised concerns about its potential health effects at these low levels of intake. To aid in the assessment of possible health risks, the extent of the actual acrylamide exposure in the general population needs to be characterized.

Acrylamide is metabolized to glycidamide by cytochrome P450 2E1 (CYP2E1). Glycidamide forms DNA adducts (Gamboa da Costa et al. 2003) and is assumed to be the agent responsible for causing the toxicologicl effects. Recent studies using CYP2E1 knockout mice have confirmed glycidamide as the actual genotoxic and reproductive toxic agent (Ghanayem et al. 2005a, 2005b). CYP2E1 is regulated at the transcriptional, translational, and posttranslational level (Ingelman-Sundberg et al. 1994) and affected by genetic polymorphisms (Itoga et al. 2002; McCarver et al. 1998). The glycidamide-to-acrylamide ratio, which can be used as an indicator of the extent of acrylamide metabolism by CYP2E1, was found to be highly variable between individuals (Bjellaas et al. 2007; Hartmann et al. 2008 Schettgen et al. 2004; Vesper et al. 2007, 2008), indicating that interindividual differences may be a factor in susceptibility to toxic effects. This high variability makes the prediction of glycidamide exposure based on acrylamide exposure difficult, and it creates the need for measuring glycidamide exposure as well as acrylamide exposure.

Hemoglobin adducts of acrylamide (HbAA) and glycidamide (HbGA) are well-established biomarkers of exposure to these chemicals (Dybing et al. 2005; Ogawa et al. 2006). The adducts are formed in a dose-dependent manner as shown in animal and human studies (Fennell et al. 2005; Tareke et al. 2006), with occupational exposures (Jones et al. 2006; Schettgen et al. 2004), with tobacco use (Kütting et al. 2008; Scherer et al. 2007; Schettgen et al. 2004; Vesper et al. 2008), and with food consumption (Hagmar et al. 2005; Wilson et al. 2008). The use of these hemoglobin adducts for exposure assessments is analogous to the use of glycated hemoglobin to monitor blood glucose levels in diabetic patients (Osterman-Golkar and Vesper 2006). HbAA and HbGA are stable over the life span of the erythrocytes, and they therefore offer a measure of cumulative internal exposure over a period of 120 days (Fennell et al. 1992).

To date, no data on internal acrylamide and glycidamide exposure acquired by use of HbAA and HbGA in the general U.S. population are available to aid in the assessment of health risks associated with this exposure. The aim of this study was to determine human exposure to acrylamide and its primary metabolite glycidamide in a representative subset of the general U.S. population.

## Materials and Methods

Specimens were obtained from participants of the 2003–2004 cycle of the National Health and Nutrition Examination Survey (NHANES), a nationally representative survey of the noninstitutionalized civilian population of the United States. NHANES obtained a stratified, multistage probability cluster sample designed to represent the U.S. population on the basis of age, sex, and race/ethnicity. Certain subpopulations were oversampled to allow for more precise estimates. All participants in the survey gave written informed consent. Information on NHANES procedures may be found at the Centers for Disease Control and Prevention Web site (CDC 2009). The NHANES protocol for each survey period was reviewed and approved by the National Center for Health Statistics Institutional Review Board.

A total of 7,166 whole-blood samples from individuals ≥ 3 years of age were used in this study. Collection was performed by antecubital phlebotomy into EDTA-containing vacutainers and stored at −70°C before analysis. HbAA and HbGA were measured in 350 μL whole blood and analyzed by high-performance liquid chromatography/tandem mass spectrometry (HPLC/MS/MS), as described previously (Vesper et al. 2007, 2008). In brief, the adducts of acrylamide and glycidamide at the N-terminal valines of hemoglobin were cleaved from the protein chain by use of modified Edman reaction with pentafluorophenyl isothiocyanate as the Edman reagent (Mowrer et al. 1986). The resulting pentafluorophenylthiohydantoin reaction products were extracted by use of liquid–liquid extraction and analyzed by HPLC/MS/MS. Calibrators, reagent blanks, and quality-control materials were processed in the same manner as the samples. Hemoglobin adduct concentrations are reported relative to the amount of hemoglobin used in the analysis. The total hemoglobin content was determined as cyanomethhemoglobin by use of a commercial assay (Stanbio Laboratory, Boerne TX). The detection limits for HbAA and HbGA adducts were 3 and 4 pmol/g hemoglobin, respectively. The interday imprecision (*n* = 20 days) of this method, expressed as percentage coefficient of variation, were 13% for HbAA and 19% for HbGA, on average, determined with three blood pools [HbAA and HbGA concentrations in picomoles per gram hemoglobin: pool 1 (135 and 93); pool 2 (103 and 700); pool 3 (62 and 50), respectively. Calibrators were synthesized by Bachem (King of Prussia, PA) from octapeptides with the same amino acid sequence as the N-terminus of the β-chain of hemoglobin reacted with acrylamide and glycidamide at the valine (AA-octapeptide: 96% purity by HPLC/UV and MALDI (matrix-assisted laser desorption/ionization)/MS; and GA-octapeptide: 90% purity by HPLC/UV and MALDI/MS). Calibrators were verified for accuracy in-house by HPLC/MS and by the U.S. Food and Drug Administration (FDA)/National Center for Toxicology Research by use of HPLC/MS and that organization’s own standards. The corresponding stable isotope-labeled compounds [AA-octapeptide and GA-octapeptide with stable-isotope labeled valine (^13^C5^15^N)] were synthesized by the same company and used as internal standards. The Edman reagent was obtained from Fluka (St. Louis, MO), and formamide was obtained from USB Corporation (Cleveland, OH). All other reagents were purchased from Fisher Scientific (Fair Lawn, NJ).

HbAA, HbGA, and cotinine levels were log10-transformed for statistical analysis, because the data distribution is skewed to the higher levels. For selected age groups, sexes, and three race/ethnicity groups, we calculated the geometric means and distribution percentiles for HbAA and HbGA, the HbGA:HbAA-ratio, and the sum of HbAA and HbGA. Race/ethnicities were self-reported as non-Hispanic black, non-Hispanic white, and Mexican American. Individuals not included in one of these three race/ethnicity groups were included in the total population estimates. Geometric means and percentiles were calculated with SUDAAN version 9.0 (Research Triangle Institute, Research Triangle Park, NC). SUDAAN uses sample weights and calculates variance estimates that account for the complex survey design. We estimated 95% confidence intervals (CIs) for geometric means were estimated on the basis of the Taylor series linearization method (Shah et al. 1996), and adapted CIs for percentiles from the methods of Korn and Graubard (1998) and Woodruff (1952). For concentrations below the limit of detection (LOD), a level equal to the LOD divided by the square root of 2 was used (Hornung and Reed 1990). Because of the known effect of smoking on acrylamide levels, we divided the data into two groups according to cotinine levels (serum cotinine ≤ 10 ng/mL: nonsmoker group; serum cotinine >10 ng/mL: smoker group) (Pirkle et al. 2006).

We calculated covariate-adjusted geometric means for selected demographic groups separately for the nonsmoking and smoking populations, using least-squares multiple regression adjusted for the covariates of age group (3–11, 12–19, 20–39, 40–59, ≥ 60 years), sex (male and female), and self-reported race/ethnicity (non-Hispanic white, non-Hispanic black, and Mexican American), and the continuous covariates of body surface area (BSA) and the base 10 logarithm of serum cotinine. We used BSA to adjust for blood volume and thus to account for other possible variability affecting delivery of acrylamide to the blood related to body size measures and was calculated according to Mosteller (1987). To arrive at the final model, we used backward elimination with SUDAAN to eliminate the nonsignificant interactions (*p* > 0.05) one at a time and then the nonsignificant main effects. Nonsignificant main effects were kept in the model as confounders when they changed the beta coefficients for significant main effects or interactions by > 10%. Once the backward procedure was completed, main effects and interactions were added back into the model one at a time and kept in the final model when they were significant (*p* < 0.05). Hypothesis tests to compare covariate-adjusted geometric means were performed using the Contrast option in the Regress procedure of SUDAAN. For further details about the final model and observed interactions between the investigated variables, see Supplemental Material, Model Information, available online (doi:10.1289/ehp.0901021.S1 via http://dx.doi.org).

## Results

**Figure 1:**
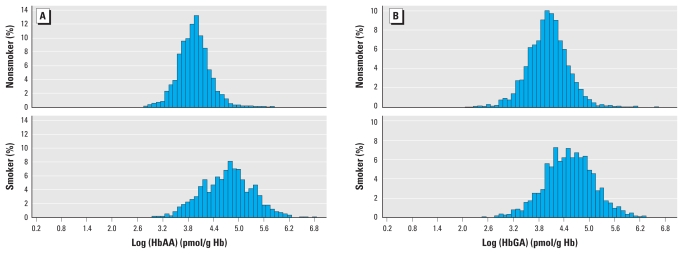
Frequency distribution of HbAA (*A*) and HbGA (*B*) in nonsmokers and smokers. Hb, hemoglobin.

HbAA and HbGA levels were detectable in 99.9% and 97.5% of all samples, respectively, with individual levels ranging from < 3 to 910 pmol/g hemoglobin for HbAA and < 4 to 756 pmol/g hemoglobin for HbGA. Distributions were different in nonsmokers and smokers (Figure 1). Geometric mean HbAA levels were 49.1–72.5 pmol/g hemoglobin higher in smokers than in nonsmokers when compared across subgroups of sex, age, or race/ethnicity (Table 1); HbGA levels were 31.6–52.2 pmol/g hemoglobin higher. Ratios of HbGA:HbAA ranged from 0.08 to 0.25 units lower in smokers than in nonsmokers when compared across subgroups of sex, age, and race/ethnicity. The intersubject variability (difference between 90th and 10th percentile) for HbAA and HbGA in nonsmokers was 45 pmol/g hemoglobin and 66 pmol/g hemoglobin, respectively [Supplemental Material, Table 1 (doi:10.1289/ehp.0901021.S1)]. In smokers, it was much larger, with 179 pmol/g hemoglobin and 191 pmol/g Hb, for HbAA and HbGA, respectively (Supplemental Material, Table 2). The subgroups of sex, age, and race/ethnicity showed similar intersubject variability as the entire group of either smokers or nonsmokers (Supplemental Material, Figures 1 and 2).

### Nonsmokers

In nonsmokers (Table 2), the covariate-adjusted geometric mean HbAA and HbGA levels were highest in children 3–11 years of age [mean, 54.5 (95% CI, 52.4–56.8) pmol/g hemoglobin for HbAA, 73.9 (95% CI, 71.3–76.6) pmol/g hemoglobin for HbGA] and lowest in adults age ≥ 60 years [46.2 (95% CI, 44.3–48.2) pmol/g hemoglobin for HbAA, 41.8 (95% CI, 38.7–45.2) pmol/g hemoglobin for HbGA]. Among the race/ethnic groups, Mexican Americans had the highest HbAA [54.8 (95% CI, 51.9–57.8) pmol/g Hb] and HbGA levels [57.9 (95% CI, 53.7–62.5) pmol/g Hb]. HbGA levels were higher in men than in women and lowest in non-Hispanic blacks [43.5 (95% CI, 41.1–45.9) pmol/g Hb] compared with non-Hispanic whites [52.6 (95% CI, 50.3–54.9) pmol/g Hb] and MA [57.9 (95% CI, 53.7–62.5) pmol/g Hb].

The HbGA:HbAA ratios were influenced by the variables of age, sex, race/ethnicity, cotinine and BSA. Several interactions were observed between these variables. Therefore covariate-adjusted geometric mean values were calculated for each age group and certain percentiles of cotinine levels by race/ethnic group [Supplemental Material, Table 3 (doi:10.1289/ehp.0901021.S1)] and for certain percentiles of BSA separate for each sex (Supplemental Material, Table 4). The ratios decreased with increasing age in all race/ethnic groups, with levels in the ≥ 60 years group [0.74 (95% CI, 0.63–0.87), 0.63 (95% CI, 0.51–0.79), 0.79 (95% CI, 0.69–0.90) in non-Hispanic whites, non-Hispanic blacks, and Mexican Americans, respectively] being approximately half of the levels in the 5–9 years group. Non-Hispanic blacks had the lowest ratios across all age groups and across all percentiles of cotinine. The relationship between non-Hispanic blacks and cotinine appears stronger than for the other race/ethnic groups. Women had higher ratios than men across all percentiles of BSA [1.05 (95% CI, 1.00–1.11) in women, 0.92 (0.87–0.97) in men, at the 75th percentile of BSA each]. The ratios decreased modestly with increasing BSA in both men and women.

Cotinine levels ranged in the nonsmoker population from 0.01 to 10 ng/mL. The estimated slope for the effect of the logarithm of cotinine levels on HbAA levels was 0.0238 (*p* = 0.0002) and on HbGA levels was 0.0228 (*p* = 0.0223), indicating that a 10% increase in serum cotinine would be associated with a 0.23% (95% CI, 0.10–0.35%) increase in HbAA levels and a 0.22% (95% CI, 0.03–0.41%) increase in HbGA levels. Simulating an increase in cotinine levels from a level equal to the first quartile (0.018 ng/mL) up to a level equal to the third quartile (0.270 ng/mL) would correspond to a 32% increase in HbAA and HbGA levels (assuming a starting point at the 10th percentile). The estimated slope for the effect of BSA in the HbAA model was –0.0399 (*p* < 0.0001), indicating that an increase in BSA from the median BSA (1.75 m^2^) to the 75th percentile of BSA (1.97 m^2^) would be associated with a 2.00% (95% CI, 1.04–2.95%) decrease in HbAA levels.

### Smokers

In smokers, HbAA and HbGA values were influenced by the variables race/ethnicity, BSA, age, cotinine, and sex. Several interactions were found between these variables. Therefore, covariate-adjusted geometric mean HbAA levels were calculated for each race/ethnic group and certain percentiles of BSA levels by sex, and the HbGA:HbAA ratio for certain levels of cotinine by sex [Supplemental Material, Table 5 (doi:10.1289/ehp.0901021.S1)]. HbAA, HbGA, and the HbAA:HbGA ratios were calculated for specific ages (20, 40, and 60 years) separately for each race/ethnic group (Supplemental Material, Table 6). Similar to nonsmokers, covariate-adjusted geometric HbGA levels were higher in females [108 (95% CI, 99.4–117)] than in males [87.9 (95% CI, 78.5–98.5)], whereas no difference was observed for HbAA levels. HbGA:HbAA ratios were higher in females than males across all percentiles of cotinine [1.59 (95% CI, 1.28–1.97) in women; 0.87 (95% CI, 0.74–1.03) in men, at the 90th percentile of cotinine each]. In addition, HbAA, HbGA levels, and the HbGA:HbAA ratios decreased with increasing age, except for Mexican Americans, where HbAA and HbGA levels remained constant. Non-Hispanic blacks had the lowest HbGA values at age groups 40 years [83.4 (95% CI, 74.8–93.1) pmol/g hemoglobin] and 60 years [59.1 (95% CI, 51.1–68.5) pmol/g hemoglobin] and the lowest HbAA/HbGA ratios across all age groups [0.45 (95% CI, 0.36–0.56) at age 60]. In contrast to nonsmokers, the HbGA:HbAA ratios decreased with increasing cotinine levels. HbAA levels modestly increased with increasing BSA in both females and males.

An increase in serum cotinine was associated with an increase in HbAA. This increase was highest at low BSA levels. A 10% increase in serum cotinine levels was associated with an increase in HbAA by 3.16% (95% CI, 2.52–3.80), 2.47% (95% CI, 1.95–2.99) and 1.93% (95% CI, 1.37–2.49) at the 25th, 50th, and 75th percentile of BSA, respectively. For HbGA, the estimated slope for the effect of the logarithm of cotinine levels was 0.1476 (*p* = 0.0001), indicating that every 10% increase in serum cotinine was associated with a 1.41% (95% CI, 0.89–1.95) increase in HbGA. A 10% increase in serum cotinine was associated with a decrease in the HbGA:HbAA ratio of 1.1% (95% CI, −1.44 to 0.76) in women and a nonsignificant decrease of 0.25% (95% CI, −0.54 to 0.005) in men.

### Relationship between HbAA and HbGA

The log-transformed HbAA and HbGA levels were linearly related (*r*^2^ = 0.4213, *p* < 0.0001). A multiple regression model for nonsmokers that included sex, race/ethnicity, cotinine, and BSA as independent variables to predict HbGA levels based on HbAA levels showed a significant interaction between race/ethnicity and HbAA levels (*p* = 0.0133). In this model, a 10% increase in HbAA was accompanied by a 9.62% (95% CI, 8.92–10.35%), 7.55% (95% CI, 6.21–8.88%), and 9.65% (95% CI, 7.96–11.36%) increase in HbGA for non-Hispanic whites, non-Hispanic blacks, and Mexican Americans, respectively. In a similar model for smokers, a 10% increase in HbAA was accompanied by an 8.62% increase in HbGA (no interactions between HbAA and the other variables were detected).

## Discussion

Exposure to acrylamide, as indicated by the presence of HbAA or HbGA, was detectable in > 99% of all NHANES participants, indicating that most of the U.S. population is exposed to this chemical. However, the levels of HbAA or HbGA were not profoundly different among several population subgroups but differed highly among individuals, suggesting that individual exposures were affected mainly by factors other than age, sex, or race/ethnicity. In addition, the relationship of levels of HbAA to HbGA could be useful in studying factors related to the metabolic conversion of acrylamide to the more toxic glycidamide.

The HbAA levels in children were modestly higher than those in two older age groups, and the HbGA levels in children were higher than those in all four older age groups even after adjustment for body size, cotinine, and sex. The higher levels in children may be due to the known larger intake of food per body mass in children, particularly acrylamide-rich foods such as french fries and potato chips, as suggested elsewhere (Dybing et al. 2005; Mucci and Wilson 2008). In addition, children have the highest HbGA:HbAA ratios, whereas adults ≥ 60 years of age have the lowest ratios. Smokers show similar patterns, with biomarker values and ratios being higher at young age. This suggests differences in the acrylamide metabolism or metabolic rate in these age groups. The reasons for these different ratios are not fully understood. Metabolism and clearance of drugs are higher in children in part because of the larger liver-to-body weight ratio and the higher blood flow through the liver compared with older adults, in whom liver volume and liver blood flow are decreased (Greim and Snyder 2008). Further investigation is needed to assess possible health effects associated with acrylamide exposure in children.

The observed differences in acrylamide exposure between race/ethnic groups, with Mexican Americans having the highest HbAA and HbGA levels and non-Hispanic blacks having the lowest HbGA levels and HbGA:HbAA ratios in nonsmokers and smokers, have not been described before. The differences observed in nonsmokers could be explained by different food consumption patterns. The 2003–2004 U.S. Department of Agriculture (USDA) food consumption survey reported differences in food consumption patterns for total sugars and carbohydrate-rich food among non-Hispanic whites, non-Hispanic blacks, and Mexican Americans (USDA-ARS 2007), and this pattern appears similar to the pattern observed for HbAA and HbGA. Carbohydrates are precursors for acrylamide formation during food processing (Mottram et al. 2002; Stadler et al. 2002). Thus, elevated consumption of carbohydrate-rich food in Mexican Americans may explain in part the elevated HbAA and HbGA levels in this group. The significantly lower HbGA:HbAA ratios and HbGA formation rate (per unit increase of HbAA) in non-Hispanic blacks may indicate differences in polymorphisms of the genes for CYP2E1 or of the genes for glutathione *S*-transferase (GST), which are involved in phase II detoxification of acrylamide and glycidamide. Different frequencies in polymorphisms in the genes for CYP2E1 in different race/ethnicity groups have been mentioned in literature (Bartsch et al. 2000; Bolt et al. 2003), and associations between polymorphisms in the genes for GST and the HbGA:HbAA ratio have been reported (Duale et al. 2009). Medications and alcohol are also metabolized by CYP2E1 and thus may affect CYP2E1 activity and metabolism of acrylamide to glycidamide. These factors could not be assessed in this study. Therefore, further studies are needed to better explain these differences in the observed HbGA:HbAA ratio.

The ranges of HbAA and HbGA levels measured in this study are similar to those reported in other study populations (Bjellaas et al. 2007; Chevolleau et al. 2007; Hartmann et al. 2008; Heudorf et al. 2008; Kütting et al. 2008; Olesen et al. 2008; Vesper et al. 2007, 2008; Wilson et al. 2008; Wirfält et al. 2008). The mean and median levels reported in these study populations for nonsmokers differ, ranging between 19 pmol/g and 47 pmol/g for HbAA and between 17 pmol/g and 49 pmol/g for HbGA. These differences in mean and median levels across different studies may be explained by differences in the demographics of the populations, differences in analytical methods used in these studies, and different acrylamide exposures in the different study populations. One recent study, investigating acrylamide exposure in groups of adults 41–60 years of age from nine European countries and using the same analytical method, found median levels among nonsmoking groups ranging between 35 pmol/g and 65 pmol/g for HbAA and 27 pmol/g and 58 pmol/g for HbGA, with the Dutch and British groups having the highest median levels (Vesper et al. 2008). The median HbAA and HbGA levels of the comparable age group in this study are similar to those found in the Dutch group (HbAA: 48.6 pmol/g hemoglobin; HbGA: 49.6 pmol/g hemoglobin).

Several investigations have attempted to compute a daily intake of acrylamide from measured levels of hemoglobin adducts (Fennell et al. 2005). In general, these are roughly comparable with intakes based on dietary estimates (Dybing et al. 2005; FDA 2006; Mucci and Wilson 2008; Svensson et al. 2003; World Health Organization 2002). Using the computation method of Fennell et al. (2005), our adduct data predict a nonsmoking overall population mean intake of 0.8 μg/kg body weight/day, which is broadly similar to some of these other estimates. Our findings indicate that secondhand smoke can increase HbAA and HbGA levels. Therefore, exposure from food may not be the only relevant exposure source in nonsmokers. Some limitations are inherent in these types of calculations, including using high-dose acute kinetic studies to compute low-dose chronic exposure estimates and difficulty in accurately accounting for additional (nonadduct) and variable pathways of acrylamide metabolism and elimination. Although such estimates provide an approximate magnitude of acrylamide exposure, further studies are needed to assess the applicability of this model for chronic exposures and for estimating subtle differences between subpopulations or exposure sources.

Because acrylamide mutagenicity depends on its conversion to glycidamide, it is of interest to determine HbGA levels and to assess the relationship between HbAA and HbGA. Therefore, we applied a multiple regression analysis to predict HbGA levels based on HbAA levels, cotinine, sex, and BSA. A 10% increase in HbAA levels was associated with an HbGA increase ranging between 7.55% and 9.65% for different population subgroups. This increase was similar for smokers and nonsmokers. Because of the high intersubject variability in acrylamide metabolism, this relationship is applicable only for population groups and cannot be applied to individuals.

The reasons for negative associations between BSA and HbAA levels in nonsmokers are not fully understood. Reported associations between body mass index and HbAA have been inconsistent (Vesper et al. 2008; Wilson et al. 2008). On the basis of observations in animal studies, Osterman-Golkar et al. (2003) suggested that increased body mass leads to an increase in blood volume and the amount of hemoglobin available for adduct formation, and thus to a dilution of the adducts at similar intake doses. This dilution effect also affects serum cotinine levels, complicating further assessments of the impact of smoking on HbAA levels, as shown in our model for smokers, where increased BSA levels tend to attenuate the effect of serum cotinine on HbAA.

The study population is representative of U.S. noninstitutionalized individuals, and the study results may not be applicable to institutionalized individuals or to other countries. A large number of statistical comparisons have been made in the analysis of the data, and some findings may be the result of random chance. The point estimates of biomarker exposure are estimates of the various subpopulation exposures to acrylamide and not estimates of individualized exposures or specific exposure sources.

## Conclusion

We have characterized U.S. population blood acrylamide and glycidamide adduct levels. Sex, age, and race/ethnicity do not strongly predict adduct levels, suggesting that the large variability in adduct levels among nonsmoking individuals is likely a result of individual intake of acrylamide from food and probably, to a lesser extent, of exposure to secondhand smoke. Dietary, metabolic, or other factors such as alcohol intake were not examined in this data set. Knowledge of all factors that affect the formation of glycidamide, as described in this study, will be important with respect to estimating risk from this known carcinogen.

## Figures and Tables

**Table 1:** Geometric mean (95% CI) (pmol/g hemoglobin) and sample size of HbAA, HbGA, and the HbGA:HbAA ratio by sex, age, race/ethnicity, and smoking status in the general U.S. population.

	Nonsmokers						Smokers					
	HbAA		HbGA		HbGA:HbAA ratio		HbAA		HbGA		HbGA:HbAA ratio	
Group	GM (95% CI)	No.	GM (95% CI)	No.	GM (95% CI)	No.	GM (95% CI)	No.	GM (95% CI)	No.	GM (95% CI)	No.
All	50.0 (48.5–51.7)	5,686	51.1 (49.0–53.2)	5,809	1.01 (0.97–1.06)	5,464	113 (103–123)	1,316	93.5 (86.5–101)	1,357	0.82 (0.79–0.85)	1,282

Sex												
Female	49.5 (48.2–50.8)	3,050	51.8 (49.1–54.7)	3,111	1.04 (0.99–1.09)	2,931	120 (108–133)	483	104 (94.9–115)	496	0.86 (0.82–0.90)	466
Male	50.7 (48.4–53.1)	2,636	50.2 (48.4–52.0)	2,698	0.98 (0.94–1.02)	2,533	108 (97.2–121)	833	87.2 (79.4–95.7)	861	0.80 (0.76–0.83)	816

Age group (years)												
3–11	58.7 (56.2–61.2)	1,070	73.4 (70.5–76.4)	1,138	1.25 (1.20–1.30)	1,030	ND^a^		ND^a^		ND^a^	
12–19	52.0 (50.1–54.0)	1,629	50.6 (46.7–54.7)	1,658	0.97 (0.89–1.05)	1,563	103 (88.1–119)	240	93.2 (80.4–108)	248	0.89 (0.85–0.93)	234
20–39	51.0 (48.5–53.8)	951	51.7 (48.4–55.3)	972	1.00 (0.95–1.06)	919	118 (106–131)	444	98.2 (88.5–109)	464	0.84 (0.80–0.88)	435
40–59	48.6 (46.1–51.2)	784	49.1 (47.0–51.2)	792	1.00 (0.95–1.06)	753	116 (106–127)	371	94.2 (87.1–102)	376	0.79 (0.75– 0.83)	362
≥ 60	44.4 (42.6–46.2)	1,252	41.0 (38.1–44.1)	1,249	0.92 (0.86–0.98)	1,199	93.5 (84.2–104)	252	77.2 (70.1–85.1)	258	0.81 (0.76–0.86)	243

Race/ethnicity												
NHW	50.1 (48.5–51.8)	2,254	51.6 (49.2–54.1)	2,287	1.02 (0.97–1.06)	2,172	114 (103–127)	698	97.8 (88.5–108)	714	0.84 (0.81–0.87)	682
NHB	50.5 (47.5–53.7)	1,480	46.3 (43.8–48.9)	1,533	0.91 (0.83–1.00)	1,405	123 (108–141)	361	82.1 (72.2–93.5)	381	0.66 (0.60–0.73)	350
MA	56.3 (53.2–59.6)	1,582	60.9 (56.8–65.3)	1,622	1.08 (1.01–1.15)	1,533	109 (98.5–120)	190	92.5 (79.7–107)	194	0.85 (0.77–0.94)	186

Abbreviations: GM, geometric mean; MA, Mexican American; ND, not determined; NHB, non-Hispanic black; NHW, non-Hispanic white.

a *n* < 10.

**Table 2:** Covariate-adjusted geometric mean estimates (95% CI) of HbAA, HbGA (pmol/g hemoglobin), and the HbGA:HbAA ratio in the nonsmoking U.S. population by sex, age, and race/ethnicity.

Parameter	Group	HbAA	HbGA	HbGA:HbAA ratio^a^
Sex	Male	51.7 (49.6–53.8)	50.1 (50.7–56.3)	0.97 (0.94–1.02)
	Female	50.3 (49.1–51.5)	53.4 (48.2–52.1)^b^	1.07 (1.03–1.13)
Age group	3–11	54.5 (52.4–56.8)^c^	73.9 (71.3–76.6)^d^	1.62 (1.39–1.87)
	12–19	51.8 (49.9–53.7)	51.9 (48.3–55.7)	1.42 (1.29–1.58)
	20–39	52.8 (50.5–55.3)	52.7 (48.8–56.8)	1.16 (1.12–1.20)
	40–59	50.7 (47.9–53.6)	50.0 (47.8–52.4)	0.86 (0.78–0.94)
	≥ 60	46.2 (44.3–48.2)^e^	41.8 (38.7–45.2)^d^	0.63 (0.51–0.78)
Race/ethnicity	NHW	50.7 (49.1–52.3)	52.6 (50.3–54.9)^f^	1.03 (0.98–1.07)
	NHB	48.9 (46.14–51.84)	43.5 (41.1–45.9)^g^	0.90 (0.82–1.00)
	MA	54.8 (51.9–57.8)^h^	57.9 (53.7–62.5)	1.06 (0.99–1.13)

Abbreviations: MA, Mexican American; NHB, non-Hispanic black; NHW, non-Hispanic white. Variables used in the model: age group, sex, race/ethnicity log-converted cotinine levels, and BSA (except for the HbGA model).

a Because of significant race/ethnicity–cotinine and sex–BSA interactions, only the levels obtained at the 50th percentile of cotinine (for race/ethnicity levels) and of BSA (for sex levels) are shown. Similarly, for age groups, the levels at the median age for each age group (7 years for 3- to 11-year group, 15.5 years for 12- to 19-year group, 29.5 years for 20- to 39-year group, 49.5 years for 40- to 49-year group, 70 years for ≥ 60-year group) are shown.

b Different from males (*p* < 0.004).

c Different from 12- to 19-year group (*p* = 0.04), 40- to 59-year group (*p* = 0.03).

d Different from all age groups (*p* < 0.0001).

e Different from 3- to 11-year group, 12- to 19-year group, 20- to 39-year group, 40- to 59-year group (*p* < 0.0001 for all groups).

f Different from MA (*p* = 0.004), NHB (*p* < 0.0001).

g Different from MA (*p* < 0.0001).

h Different from NHW (*p* < 0.008), NHB (*p* < 0.0001).
